# An Ebola virus-centered knowledge base

**DOI:** 10.1093/database/bav049

**Published:** 2015-06-08

**Authors:** Maulik R. Kamdar, Michel Dumontier

**Affiliations:** Stanford Center for Biomedical Informatics Research, Department of Medicine, Stanford University, Stanford, CA, USA

## Abstract

Ebola virus (EBOV), of the family *Filoviridae* viruses, is a NIAID category A, lethal human pathogen. It is responsible for causing Ebola virus disease (EVD) that is a severe hemorrhagic fever and has a cumulative death rate of 41% in the ongoing epidemic in West Africa. There is an ever-increasing need to consolidate and make available all the knowledge that we possess on EBOV, even if it is conflicting or incomplete. This would enable biomedical researchers to understand the molecular mechanisms underlying this disease and help develop tools for efficient diagnosis and effective treatment. In this article, we present our approach for the development of an Ebola virus-centered Knowledge Base (Ebola-KB) using Linked Data and Semantic Web Technologies. We retrieve and aggregate knowledge from several open data sources, web services and biomedical ontologies. This knowledge is transformed to RDF, linked to the Bio2RDF datasets and made available through a SPARQL 1.1 Endpoint. Ebola-KB can also be explored using an interactive Dashboard visualizing the different perspectives of this integrated knowledge. We showcase how different competency questions, asked by domain users researching the druggability of EBOV, can be formulated as SPARQL Queries or answered using the Ebola-KB Dashboard.

**Database URL:** http://ebola.semanticscience.org.

## Introduction

Ebola virus (EBOV) is a member of the species *Zaire ebolavirus* and is one of the five known viruses within the genus *Ebolavirus*. The other members of this genus are Bundibugyo virus (BDBV), Reston virus (RESTV), Sudan virus (SUDV) and Taï Forest virus (TAFV) (1). Four of these viruses (BDBV, EBOV, SUDV and TAFV) cause a severe and often fatal hemorrhagic fever, called Ebola virus disease (EVD), in humans. EBOV and SUDV were responsible for the 1976 EVD epidemic in Northern Zaire and Southern Sudan, respectively (2). BDBV was discovered during the 2008 EVD outbreak in Uganda (3). TAFV was documented to cause EVD in a single, isolated case in Ivory Coast, West Africa in November 1994 (4). EBOV, the most lethal of the known EVD-causing viruses, has caused the majority of EVD outbreaks and is responsible for the 2013–2015 manifestation of EVD that has a cumulative death rate of 41% (as of 20 March 2015) among confirmed, probable and suspected cases in West Africa (5). EBOV has an enveloped, single-stranded, nonsegmented, negative-sense ribonucleic acid (RNA) genome which is very similar in organization to viruses within *Marburgvirus* [Marburg Virus (MARV) and Ravn Virus (RAVV)]. *Ebolavirus* (BDBV, EBOV, RESTV, SUDV and TAFV) and *Marburgvirus* (MARV and RAVV) constitute a part of the family *Filoviridae* (1). These viruses have been classified as Tier-1 Select Agents, World Health Organization (WHO) Risk Group 4 Pathogens, National Institutes of Health/National Institute of Allergy and Infectious Diseases (NIH/NIAID) Category A Priority Pathogens, and Centers for Disease Control and Prevention (CDC) Category A Bioterrorism Agents. Whereas most of the EVD outbreaks (6) were confined to remote regions of Central Africa and had minimal deaths, the ongoing EVD epidemic, which began in Guinea in December 2013, has spread exponentially across five other countries in Western Africa and more than 24 000 cases have been reported (as of 20 March 2015) cumulatively in a short period of time (5). The 41% cumulative death rate is likely to be a substantial underestimate of the actual case fatality rate, as it does not account for the delay between onset of EVD symptoms and outcome (i.e. recovery or death) and includes probable and suspected cases also (7, 8).

The Viral Hemorrhagic Fever Consortium sequenced a set of 99 EBOV sequences from 78 confirmed patients in Sierra Leone to 2000× coverage [BioProject: PRJNA257197 (9)] (10). Due to the largest EVD epidemic in history, there is a dire need to consolidate and integrate all available knowledge that we currently possess or could be retrieved from open-access knowledge bases and available literature, on the EBOV genome. This could lead to a better understanding of the underlying mechanisms of EVD, determination of the druggability of target domains in EBOV and identification of small molecules which could show positive binding affinity. However, as EVD was not considered a high priority threat before the start of this ongoing epidemic, information pertaining to the above goals is not yet available at a single, aggregated source. As a consequence, the biomedical researcher has to traverse across several data portals to retrieve relevant knowledge before using it for the formulation of hypotheses that could lead to drug repurposing or diagnosis for EVD.

The challenges stemming from the integration of disparate, heterogeneous life sciences datasets on the web has led to the adoption of a new set of technologies, based on the Semantic Web and Linked Data concepts, by the biomedical researchers (11). The Semantic Web provides a common framework that facilitates data representation in machine-processable formats and data sharing and reuse across application, enterprise, and community boundaries (12). Linked Data concepts refer to a set of best practices, adopted by numerous data providers, for publishing and connecting structured data on the web, leading to the creation of a global web of data comprised of billions of assertions (13). These technologies have facilitated universal named entity disambiguation, reasoning, retrieval and aggregation of information pertaining to a certain gene, disease or protein from multiple data sources simultaneously (14, 15). Biomedical communities engaged in the Linked Data efforts have emphasized the need for publishing biomedical resources using the Resource Description Framework, a World Wide Web Consortium (W3C) Recommendation for publishing data on the web in a machine understandable manner (16). They have exposed the associated data sources through SPARQL query endpoints, and have also agreed on a concise set of ontologies and standards for exchanging experimental data and biomedical information (17).

In this article, we present the approach used towards the construction of an Ebola virus-centred Knowledge Base (Ebola-KB). We retrieve, transform and integrate information pertaining to the Ebola Virus from multiple sources and link it to existing Bio2RDF datasets (14), leveraging knowledge discovery. In Linked data section, we describe briefly the Bio2RDF project and its application to retrieve meaningful information from various, heterogeneous life sciences datasets for practical use cases. In Materials and methods section, we delve deeper into the different types of data integrated into the Ebola-KB, and the methods used to aggregate this data from their respective sources. In Applicability section, we demonstrate a web-based interface facilitating interactive ease-of-access of the Ebola-KB for domain users. We showcase the formulation of few competency questions, asked by the biomedical researchers trying to gather existing knowledge on EBOV, as SPARQL queries. In Discussion section, we discuss the limitations of our approach and the application of this knowledge to understand the molecular mechanisms underlying EVD towards assessing the druggability of EBOV.

## Linked data

Several key initiatives in the past decade have led to the formal representation of biomedical datasets on the web and integration across these diverse data sources through identification using Unique Resource Identifiers (URIs). Bio2RDF, an open-source project, uses Semantic Web technologies to build and provide the largest network of Linked Data for the Life Sciences (14). Bio2RDF defines a set of simple conventions to create RDF(S) compatible Linked Data from a diverse set of heterogeneously formatted sources obtained from multiple data providers. The Bio2RDF Release 3 consists of around 11 billion triples generated from 35 important biomedical data sources (18). These RDF datasets are exposed for real-time querying over HTTP using the SPARQL 1.1 protocol.

The utility of Bio2RDF has been previously demonstrated to generate a mashup of microarray gene expression results with interaction data obtained from the HIV-1, Human Protein Interaction Database (HHPID) (19) to understand the infection of human macrophages with human immunodeficiency virus 1 (HIV-1) (20). Over time, Bio2RDF has been cross-linked with several other data sources, including a chemogenomic and chemical systems biology data repository (Chem2Bio2RDF) (21) and a Semantic Web Atlas of post-genomic knowledge on mouse and human (22), and has been used to develop Linked Biomedical Dataspaces facilitating *in silico* drug discovery in specialized domains (23, 24). Hence, exposing EBOV knowledge as a Bio2RDF knowledge base could help biomedical researchers discover newer insights into EBOV, examine the druggability of EBOV and lead to possible treatment for EVD.

## Materials and methods

### Identification of conserved regions and associated gene ontology terms

The EBOV RNA genome consists of between 18 956 and 18 961 nucleotides (25) and encodes of 9 distinct, known protein sequences (26, 27), some of which have similar protein domains with other viruses of the *Filoviridae* family. The National Centre for Biotechnology Information (NCBI) portal (28) exposes various biological databases like the GenBank nucleic acid sequence database (29), PubMed (30) and the BioProject database (31), and also provides tools for retrieval and analysis of the data. The results of the EBOV sample sequencing analysis, conducted by the Viral Hemorrhagic Fever Consortium, were retrieved from the NCBI portal (10). The metadata of the nucleotide and protein resources were downloaded from the BioProject database as GenPept files, whereas the sequences as aggregated under the BioProject PRJNA257197 (9) were retrieved from GenBank in the FASTA format.

In order to identify putative biological functions of the EBOV genes and protein-binding small molecules, it is first necessary to determine the functional sites and domains of the encoded proteins. InterPro (32) is an integrated documentation resource for protein families, domains and functional sites, which combines several protein signature databases like Pfam (33), PROSITE (34) and Phobius (35), for identifying relationships in novel protein sequences and inferring protein function. We use the InterProScan API (36) for scanning the protein sequences generated against the protein signatures in the member databases of InterPro. Each InterPro result is also annotated with Gene Ontology (GO) (37) terms describing the associated molecular function, biological process or cellular component of the protein signature. The API is queried using the EBOV protein sequences as parameters and the conserved relationships and GO terms are retrieved. For each GO Term, we query the EBI QuickGO Web Services (38) to obtain GO Term information and other similar domains annotated with the same term, indicating a shared function, process or localization.

### Integration of PubMed articles

Due to the ongoing EVD epidemic, there is a lot of experimental, wet-laboratory research being conducted to understand the mechanisms of molecular action of the EBOV as well as to assess the potential druggability of target regions. To keep our knowledge base current and updated of this ongoing research, we decided to retrieve and integrate the metadata (title, authors, abstract, etc.) of relevant publications. PubMed (30) is an online repository, which comprises of more than 24 million citations for biomedical literature from MEDLINE (39), life science journals, and online books. We extracted a set of EBOV-related keywords from the protein, domain and GO terms retrieved in the previous task and searched the PubMed Database for articles tagged with those keywords. We used the Entrez Programming Utilities (E-utilities) API (40) exposed by the NCBI for text search and retrieve the associated PubMed IDs. We then retrieve the full metadata record of the article in XML format by iteratively querying the E-utilities API using the IDs.

### Retrieval of putative domain-binding small molecules

To determine the small molecules which could bind EBOV protein domains, and lead to potential drug repurposing, it is important to first provide the domain researcher with a list of existing ligands which have been proven to bind similar domains. These ligands could then be filtered using a set of predefined criteria and *in silico* computational analysis, before testing them *in vivo*. The Protein Data Bank (PDB) is an archive consisting of three-dimensional structural data on biological molecules, and their interactions with ligands, as detected by various experimental methods (41). PDB is updated very frequently and also contains the structural information and metadata of putative small molecule ligands which inhibit key functions of the EBOV proteins as determined by several ongoing experiments using *in silico* methods like NMR Spectroscopy, complex X-ray diffraction and crystallography (42). We query the PDB REST (43) Text Search API using EBOV Keywords generated from the EBOV protein and domain descriptions previously to retrieve a list of associated PDB IDs, and iteratively call their Ligand Search API for getting the metadata (chemical name, formula, SMILES Notation, Molecular Weight, Inchi Keys etc.) of individual ligands in the structural interactions, in XML format. We also catalogue the provenance information of the underlying experiments that registered the interactions.

We were further interested to associate knowledge on targets, pathways implicated, action mechanisms and activities for some of these ligands, which were existing compounds used to inhibit other protein targets. In particular, we decided to integrate two knowledge bases—DrugBank, a comprehensive resource for protein targets and drug action information used in *in silico* drug discovery (44), and Kyoto Encyclopedia of Genes and Genomes (KEGG), a knowledge base linking gene functions and genomic features to higher order molecular pathways (45). To integrate Ebola-KB with the web of biomedical Linked Open Data and enable the domain researcher to obtain additional information, like pharmacology, action mechanism, different packagers of the binding ligands, we query the Bio2RDF DrugBank dataset using their InChI Keys and determine the DrugBank URI and label of the corresponding small molecule which has a similar value for the InChI Key property.

### Data conversion to RDF and linking to existing Bio2RDF datasets

**Figure 1.**
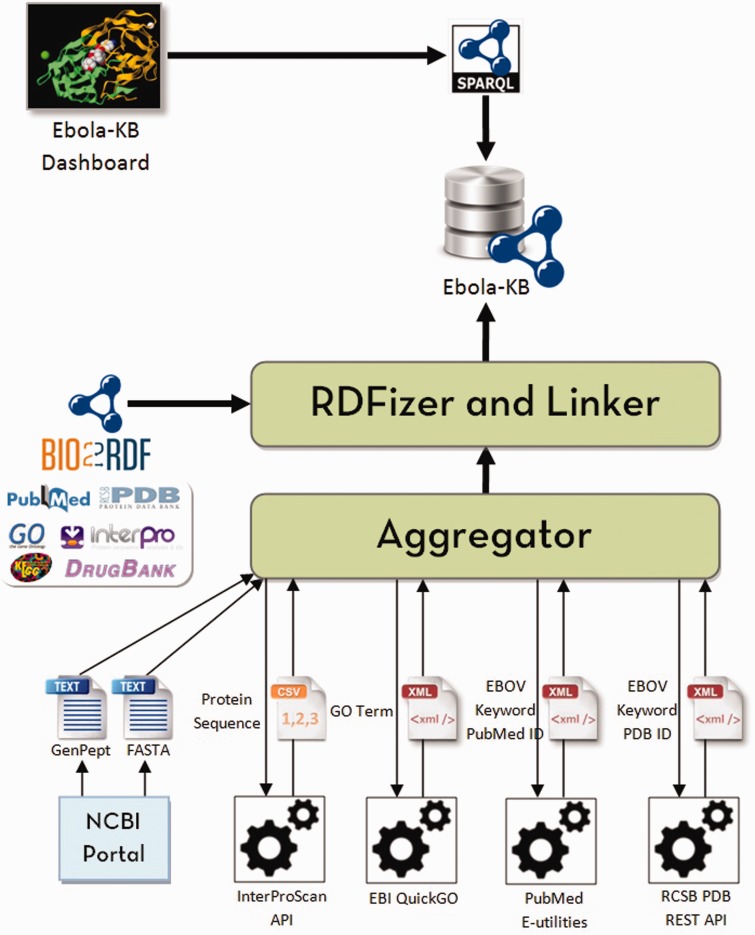
System architecture to build and update the Ebola-KB. The Aggregator queries the Web APIs to aggregate data from the underlying data sources (InterPro, GO, PubMed and PDB). The RDFization module uses Open Refine and the Ebola-KB Vocabulary to transform the aggregated data to RDF. We finally link the retrieved entities (genes, domains, PDB Structures, GO Terms, publications and ligands) to similar entities in Bio2RDF datasets (NCBIGene, InterPro, PDB, GO, PubMed and DrugBank respectively). The Ebola-KB is exposed as a SPARQL 1.1 endpoint which can be queried by the Ebola-KB dashboard.

**Figure 2.**
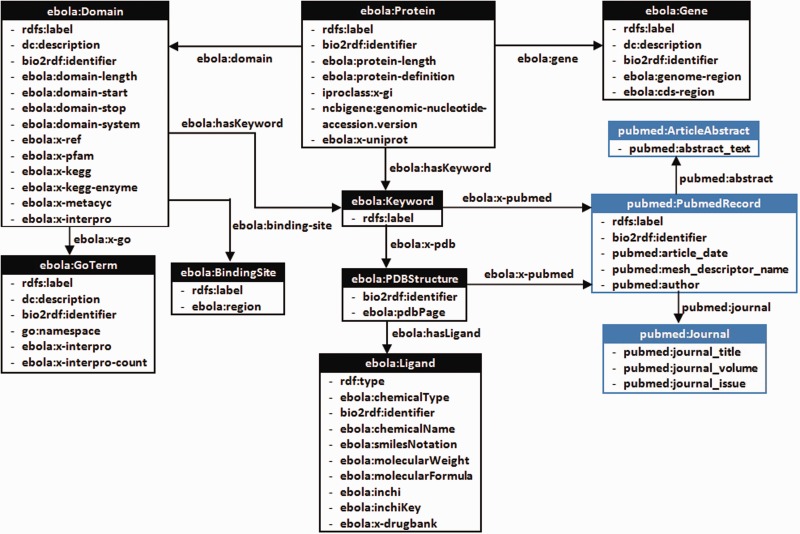
A class diagram of the custom-developed Ebola-KB Vocabulary (Supplementary Appendix III) for transforming the aggregated data into RDF. We reuse elements from the Bio2RDF PubMed Vocabulary (highlighted in blue), and provide properties ebola:x-ref and ebola:x-<database> to link back to associated entity URIs from other data sources.

Our primary objective is to aggregate all the existing information pertaining to the EBOV genome from various biomedical data sources into a single knowledge base and provide means for integrated access to knowledge from other databases using a single interface to enable knowledge-based discoveries. We decided to use Linked Data principles and Semantic Web technologies to transform the aggregated information to a graph-based representation using RDF and link the entities (genes, proteins, domains, publications and ligands) to the corresponding resources in Bio2RDF datasets using explicitly typed-relationships. The system architecture used to build and update the Ebola-KB is shown in Figure 1. An Aggregator module queries the aforementioned Web APIs to retrieve specific information related to the EBOV genome from the underlying data sources in varied formats. The RDFization module uses a custom-developed Ebola-KB vocabulary for transforming the aggregated data to RDF. The different classes and properties taken into consideration are shown in Figure 2. Information retrieved from the EBOV genome sequencing analysis is modelled using the *ebola:Gene*, *ebola:Protein*, *ebola:Domain* and *ebola:BindingSite* classes. GO Terms are cataloged under the *ebola:GoTerm* class. Keywords extracted from the protein, domain and GO Terms are listed as instances of the *ebola:Keyword* class and linked to the associated entity using *ebola:hasKeyword* object property. Data retrieved using the RCSB PDB REST API is modelled as instances of the *ebola:PDBStructure* and *ebola:Ligand* classes, using the extensive set of datatype properties for the *ebola:Ligand* class.

Besides publishing data on the web as RDF, the Linked Data Design principles encourage use of HTTP de-referenceable URIs, vocabulary re-use and provision of links to other data sources (13). This facilitates cross-ontology question answering, inference and data integration. Hence, we decided to re-use elements from the PubMed Vocabulary, namely *pubmed:PubMedRecord*, *pubmed:Journal* and *pubmed:ArticleAbstract* (highlighted using blue colour). For this work, we integrate the Bio2RDF InterPro, PDB, NCBIGene, PubMed, GO, KEGG and DrugBank datasets, either by using the same URIs or linking the corresponding resource URIs using explicitly typed relationships of the form *ebola:x-ref* and *ebola:x-<database>*. For example, the Bio2RDF DrugBank URIs retrieved from matching the Inchi Keys of the PDB ligands with the DrugBank small molecules are linked using the *ebola:x-drugbank* property. Hence, Ebola-KB is connected to the Biomedical Linked Open Data Cloud (46), and information contained in other RDF stores connected through these intermediate Bio2RDF nodes could also be accessed by the researcher. The different classes and properties of the Ebola-KB RDF Vocabulary are presented under Supplementary Appendix III.

We use a batch process to clean and transform the information to RDF using the RDF Refine extension (47) of the Open Refine workbench. These scripts can be automated using regular cron jobs for keeping our knowledgebase updated. The generated RDF graphs are uploaded to an OpenLink Virtuoso instance, and exposed as a SPARQL query endpoint.

## Applicability

The Ebola Knowledge Base (Ebola-KB) has been made available at http://ebola.semanticscience.org/. The RDF datasets, SPARQL Endpoint and the end-user application can be accessed directly from here.

### Ebola-KB dashboard

**Figure 3.**
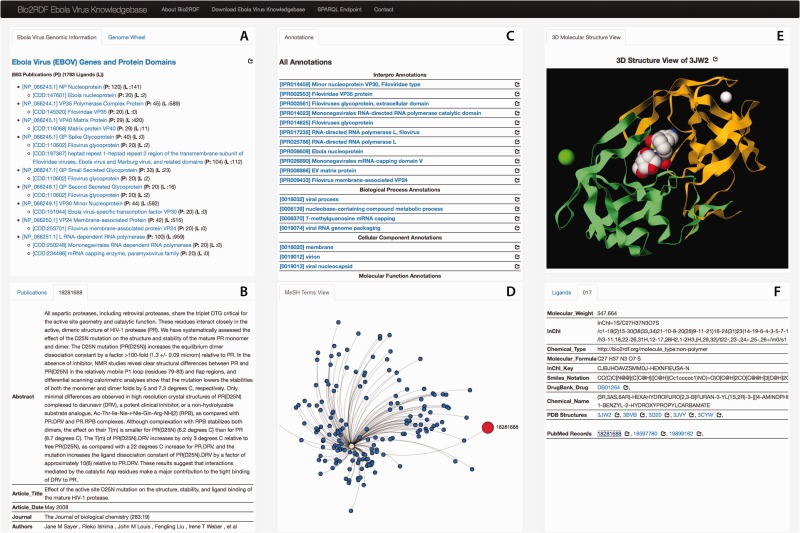
Ebola-KB Dashboard: (**A**) Summarized List of the EBOV Genes and Protein Domains, and the EBOV Genomic Wheel, (**B**) Publications associated with a genomic region and additional information for any selected publication, (**C**) InterPro and Gene Ontology Annotations, (**D**) MeSH Terms View, (**E**) 3D-Molecular structure View for PDB entities and (**F**) Associated Ligands and additional information on any selected ligand.

To facilitate easy access to the Ebola-KB, we developed a data summarization and visualization tool, the Ebola-KB Dashboard that presents all the information in the Ebola-KB through interactive widgets. As shown in Figure 3, the Dashboard consists of six primary widgets, providing different views of the information categorized in the Ebola-KB. The Widget A provides a summarized representation of the EBOV genes denoted using the consensus EBOV RefSeq entry (48, 49), and the underlying protein domains listed in the Conserved Domain Database. The total number of publications and ligands associated with any particular genomic region are also shown.

**Figure 4.**
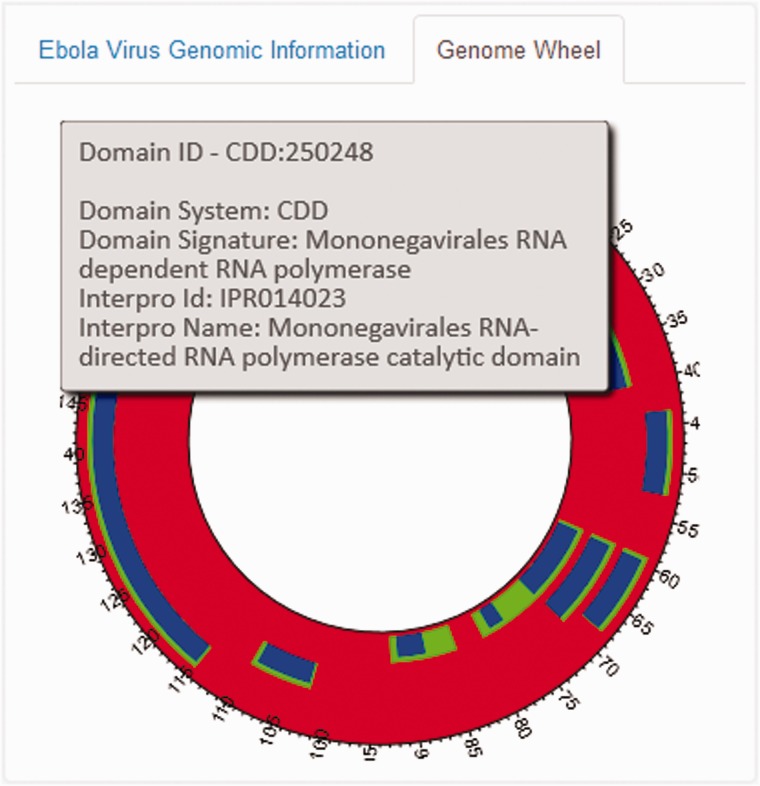
The EBOV Genome Wheel visualizes the spatial organization of the genomic regions.

An EBOV Genome Wheel (50) aids in the visualization of the spatial organization of the genomic regions (Figure 4). The Widgets B, C and F show the list of PubMed publications, InterPro and Gene Ontology annotations and PDB ligands associated with any genomic region. We also offer provenance information by providing an external reference to the main source. Clicking on any particular entity shows additional information pertaining to that entity. For instance, as shown in Figure 3B, the metadata (title, journal information, abstract, authors and MeSH Terms) of the PubMed record as retrieved from their E-utilities API or the additional information on the clicked ligand (chemical name, molecular weight, molecular formula, InChI and SMILES Notation) is shown in the widgets. We also provide a widget which visualizes the PubMed records as a force-directed network graph linked to their MeSH terms, for enabling quick access to related publications with similar MeSH annotations. Finally, Widget E displays a three-dimensional structure visualization of PDB structures which contain the ligands.

The Ebola-KB Dashboard uses modern JavaScript libraries for rendering HTML5 Canvas visualizations. The MeSH Terms View employs the SigmaJS (51) and the Force-Atlas graph layout algorithm (52). We used the KineticJS library (53) for the EBOV Genome Wheel and WebGL-based GLMol Molecular Viewer (54) for the 3D-Structural View. The Ebola-KB Dashboard can be downloaded and deployed locally using an Apache Server with PHP5 and PHP-CURL support enabled. The platform communicates with the Ebola-KB endpoint using the SPARQL 1.1 protocol and retrieves the results in JSON format.

### Use cases

#### Identify the biological activities and associated scientific publications of the EBOV gene product *‘Polymerase’*

The initial step towards ascertaining the druggability of different EBOV genes is to identify the conserved domains of the encoded protein, the biological processes or molecular functions associated with the gene and any available knowledge in literature on the gene or its products. Without the use of Linked Data technologies, this would entail a requirement to query the individual data repositories and combine the information manually. The methodology used by us to build the Ebola-KB, as shown in the aforementioned section, enables a biomedical researcher to retrieve this information in an aggregated fashion. As shown in Supplementary Appendix I (Listing 1), he can build a SPARQL CONSTRUCT which combines the retrieved Gene Ontology Annotations and metadata of scientific publications associated with the *‘Polymerase’* gene as a graph.

Moreover, the Ebola-KB Dashboard makes this information just a click away from the biomedical researcher. The user of the Dashboard can click on the desired gene *‘[NP_066251.1] RNA-dependent RNA Polymerase’*, and the adjoining widgets are automatically updated to facet on this selection, and show only the associated publications and annotations from an initial listing. The user can then click on any particular publication to read its abstract and other information in the adjoining tab.

#### Retrieve knowledge from KEGG and DrugBank on small molecule ligands which bind to EBOV protein *‘Polymerase’*

After reviewing the literature and the biological activities associated with the EBOV gene *‘Polymerase’*, the researcher would now like to proceed on the next step of his analysis. He would like retrieve knowledge available in KEGG and DrugBank on all the putative small molecule ligands which may bind to protein product of *‘Polymerase’* or other similar proteins, as determined by different *in silico* experiments (NMR Spectroscopy, X-ray diffraction, etc.). He could formulate the SPARQL CONSTRUCT Query as shown in Supplementary Appendix I (Listing II and III), which uses a Sub-Select SPARQL query against the respective SPARQL endpoint of Bio2RDF KEGG and DrugBank. Information like chemical name, molecular formula, molecular weight, SMILES notation of the ligands contained within PDB structures associated with *‘Polymerase’* can also be obtained. Using the Ebola-KB Dashboard, clicking on the relevant gene constrains the list of ligands automatically. The properties of each ligand entry in the list can then be viewed in the separate tab, or the three-dimensional PDB structure can be visualized (Figure 3E and F).

Using the subselect queries, we obtain the pharmacology, action mechanism and packagers of these set of ligands from DrugBank, and activity, drug targets and implicated pathways from KEGG using the kegg:x-drugbank predicate. The aggregated RDF graphs can be combined to create an exemplary data table as shown in Supplementary Appendix II, to aid towards re-purposing of existing drugs against EBOV protein products. It can be seen that we obtain small molecules with varied activities ranging from *‘Analgesic’*, *‘Anticancer’*, *‘Antibacterial’* and *‘Antiviral’* (which was desired), however on individual inspection of each corresponding PDB entry, we found that they were related to our initial EBOV search term *‘Polymerase’*.

#### Obtain scientific publications that provide evidence for the binding of the ligands ‘Rifampicin (RFP)’, ‘Rifabutin (RBT)’ and ‘Rifapentine (RPT)’

After retrieving the set of putative small molecule ligands which bind to the EBOV protein *‘Polymerase’*, the researcher would like to know which scientific publications provide evidence to the binding of these ligands to other similar proteins or domains. Before starting *in vivo* experiments for the druggability of the EBOV domain using the binding ligand, this step is relatively important for the domain researcher. The researcher may be interested to determine if the ligand under question is also effective against wild type or rapidly mutating strains, and does not bind to other unrelated human proteins causing adverse side effects. For instance, after checking the exemplary table retrieved in the previous step, the researcher may be interested to investigate further into the drugs ‘Rifampicin (RFP)’, ‘Rifabutin (RBT)’ and ‘Rifapentine (RPT)’, which fall under the KEGG category of *‘Rifamycins’*, are known to target *bacterial DNA-Dependent RNA Polymerase* (55)*,* and are clinically approved for treatment in Tuberculosis. During our data collection phase, we had also stored the provenance information for the underlying experiments which registered the interactions between a binding ligand and its target domain.

The relevant SPARQL query is shown in Listing 4 in Supplementary Appendix I. On the execution of the SPARQL query, a brief glance on the metadata of the retrieved PubMed records shows intricate details and minute differences of the mode of actions of different *‘Rifamycins’*—e.g. contrary to *‘Rifampicin’* and *‘Rifapentin’*, *‘Rifabutin’* inhibits formation of the first and second phosphodiester bonds and may have superior affinity for wild-type and rifampin-resistant *Mycobacterium tuberculosis* (56). The scientific evidence can be accessed using the Dashboard, where the tab showing additional information on a selected ligand, also lists the associated PubMed IDs, which can be clicked to view the publication record (Figure 3B and F).

## Discussion

### Challenges and limitations

As EVD was not considered a high research priority until now, there is lack of integrated knowledge pertaining to gene functions, protein interactions, available literature and activities of binding ligands. However, to counter the ongoing EBOV-caused EVD epidemic, it is imperative for the biomedical researcher to gain insights into the molecular mechanisms underlying EVD for efficient diagnosis, examine the druggability of EBOV genomic regions and conduct *in vivo* analysis for concise set of binding small molecules for effective therapy. Our vision for developing Ebola-KB was that it would provide a starting point to the researcher to access the heterogeneous data from multiple data sources through a single interface. Hence it involved the aggregation of the aforementioned knowledge.

It was however difficult as some of the popular knowledge-bases like STITCH, the resource for chemical–protein interaction networks (57), did not provide any information on the small molecules which bind the EBOV protein sequences, or those binding other similar proteins. Moreover, we also found that very few EBOV InterPro domains had actually been annotated with Gene Ontology Terms. Our approach to generate and use EBOV Keywords as search terms for PDB and PubMed, allowed us to expand the search space, but the automated pipeline also incorporated some ‘noise’ in the Ebola-KB, e.g. information on ligands binding ‘*DNA Polymerase’* in species. Hence, manual curation was also required. More rigorous protein and domain-similarity features could be used in the future to tackle this. Our use of Semantic Web Technologies (RDF, SPARQL) would allow us to deal with the heterogeneity of the data sources and provide integrated access across several biomedical data sources, as shown in the KEGG/DrugBank use case, but it also necessitates the availability and better uptime of SPARQL Endpoints, which may not always be the case (24).

### Future work

We would like to provide a curation feature in the Ebola-KB Dashboard for authenticated users and a RDF-versioning framework in the backend to keep Ebola-KB updated with the primary data sources. It would be interesting to include the rich information on BioAssays and activities of small molecules binding potential virus targets, which is available on PubChem (58), as well as the full text publications archived in PubMed Central (59), with the Ebola-KB. PubChem information related to the EBOV genome could be retrieved by querying the NCBI E-utilities with specific EBOV keywords, as shown in our integration approach from PubMed. We want to convert all available genome sequences of *Z.* *ebolavirus* on the NCBI Portal to RDF and associate each sequence to the publications that mention it through text mining. We hope to delve into methods which help predict small molecule binding sites on proteins with a known or unknown structure, given a protein sequence. An expansion of SMID-BLAST (Small Molecule Interaction Database) could be considered (60). Mouse Model Phenotypes could be used to study the binding profiles of the aggregated small molecules in Ebola-KB against the EBOV drug targets (61). We have started carrying out extensive outreach activities as presentations and poster sessions to make our target audience aware of this aggregated knowledgebase, as well as delving into newer use cases for linking other useful data sources. We plan to carry out a user-driven evaluation to gauge the intuitiveness of the Ebola-KB Dashboard for the domain users.

## Conclusion

In the ongoing epidemic in Western Africa, EVD has reportedly infected more than 24 000 people, with a cumulative death rate of 41% (as of 20 March 2015) (5). As a result, it is desirable to consolidate all the knowledge pertaining to Ebola Virus (EBOV) genome, whether proven or conflicting, and make it available through a single portal. In this report, we have showcased our methodology to create an Ebola virus-centered Knowledge Base (Ebola-KB), using Semantic Web Technologies and Linked Data principles. We have laid out a roadmap for the creation of a semiautonomous pipeline which queries and aggregates knowledge from the InterProScan, EBI QuickGO, PubMed E-utilities and RCSB PDB REST APIs, and combine it with the 2014 EBOV genome sequencing results downloaded from the NCBI Portal. We transform this retrieved knowledge to RDF and link them to the relevant resources in Bio2RDF repositories NCBIGene, PubMed, InterPro, GO, PDB, DrugBank and KEGG using URIs. The Ebola-KB has been exposed as a SPARQL 1.1 endpoint for querying, and an interactive Dashboard is also provided which visualizes the different perspectives of the underlying knowledge and allows the biomedical researcher to selectively isolate desired information. We have shown three use cases on how the Ebola-KB and the Dashboard can be used by domain users to investigate the molecular mechanisms underlying EVD and explore the druggability of the EBOV genome.

## Supplementary Data

Supplementary data are available at *Database* Online.

## Funding

Stanford University start-up fund (M.D.) Funding for open access charge: XXX.

*Conflict of interest*. None declared.

## Supplementary Material

Supplementary Data
