# Supplementary material for: An Ebola virus-centered knowledge base
Source: Database (Oxford). 2015 Jun 8;2015:bav049. doi: 10.1093/database/bav049 (PMC4460400; doi:10.1093/database/bav049)
Supplement: Supplementary Data [file supp_2015_bav049_index.html]

An Ebola virus-centered knowledge base — Supplementary Data 

# An Ebola virus-centered knowledge base

## Supplementary Data

files

- Supplementary Data - zip file
