# Supplementary material for: An Ebola virus-centered knowledge base
Source: Database (Oxford). 2015 Jun 8;2015:bav049. doi: 10.1093/database/bav049 (PMC4460400; doi:10.1093/database/bav049)
Supplement: Supplementary Data [file supp_bav049_suppl_data.zip › Figure legends.docx]

**Figure 1:** System architecture to build and update the Ebola-KB. The Aggregator queries the Web APIs to aggregate data from the underlying data sources (InterPro, GO, PubMed and PDB). The RDFization module uses Open Refine and the Ebola-KB Vocabulary to transform the aggregated data to RDF. We finally link the retrieved entities (genes, domains, PDB Structures, GO Terms, publications and ligands) to similar entities in Bio2RDF datasets (NCBIGene, InterPro, PDB, GO, PubMed and DrugBank respectively). The Ebola-KB is exposed as a SPARQL 1.1 endpoint which can be queried by the Ebola-KB dashboard.

**Figure 2:** A class diagram of the custom-developed Ebola-KB Vocabulary (Appendix III) for transforming the aggregated data into RDF. We re-use elements from the Bio2RDF PubMed Vocabulary (highlighted in blue), and provide properties ebola:x-ref and ebola:x-<database> to link back to associated entity URIs from other data sources.

**Figure 3:** Ebola-KB Dashboard: A) Summarized List of the EBOV Genes and Protein Domains, and the EBOV Genomic Wheel, B) Publications associated with a genomic region and additional information for any selected publication, C) InterPro and Gene Ontology Annotations, D) MeSH Terms View, E) 3D-Molecular structure View for PDB entities, and F) Associated Ligands and additional information on any selected ligand.

**Figure 4:** The EBOV Genome Wheel visualizes the spatial organization of the genomic regions.
