# Supplementary material for: An Ebola virus-centered knowledge base
Source: Database (Oxford). 2015 Jun 8;2015:bav049. doi: 10.1093/database/bav049 (PMC4460400; doi:10.1093/database/bav049)
Supplement: Supplementary Data [file supp_bav049_suppl_data.zip › Appendix I.docx]

**Appendix I - SPARQL Queries**

| PREFIX ebola: <<http://bio2rdf.org/ebola_vocabulary>:> PREFIX go: <[http://www.geneontology.org/go#](http://www.geneontology.org/go)> PREFIX pubmed: <<http://bio2rdf.org/pubmed_vocabulary>:>   PREFIX graph: <<http://localhost:8080/meta_graph>:>  PREFIX xsd: <http://www.w3.org/2001/XMLSchema#>                                      CONSTRUCT {     <http://bio2rdf.org/refseq:NP_066251.1> graph:domain ?interprolabel;  graph:go-uri ?goTerm; graph:pubmed-uri ?pubmedUri .     ?goTerm rdfs:label ?golabel; go:namespace ?namespace.     ?pubmedUri rdfs:label ?pubmedTitle;  graph:journal-metadata `bif:concat (?journalTitle, " (", ?journalVol, ":", ?journalIssue, ")")` ;     graph:abstract ?abstractText; graph:article-date ?articleDate;  graph:author ?author; graph:mesh-term ?mesh } WHERE {     ?protein ebola:domain ?domain.  ?protein ebola:protein-definition ?proteindefn.     ?domain ebola:x-interpro ?interproDomain .  ?interproDomain rdfs:label ?interprolabel.     OPTIONAL {?domain ebola:x-go ?goTerm .  ?goTerm go:namespace ?namespace; rdfs:label ?golabel}     ?protein ebola:hasKeyword ?keyword .     ?keyword ebola:x-pubmed ?pubmedUri .     ?pubmedUri rdfs:label ?pubmedTitle; pubmed:abstract ?abstract; pubmed:journal ?journal.     ?journal pubmed:journal_title ?journalTitle .     ?abstract pubmed:abstract_text ?abstractText .     OPTIONAL {?journal pubmed:journal_volume ?journalVol }     OPTIONAL {?journal pubmed:journal_issue ?journalIssue}     OPTIONAL {?pubmedUri pubmed:article_date ?articleDate}     OPTIONAL {?pubmedUri pubmed:author ?author}     OPTIONAL {?pubmedUri pubmed:mesh_descriptor_name ?mesh}  FILTER( regex( xsd:string( ?proteindefn ), "polymerase", "is" ) ) . } |
| --- |
| **Listing 1:** SPARQL CONSTRUCT Query to retrieve a graph of InterPro and Gene Ontology annotations and metadata of all the associated PubMed publications for the EBOV *‘Polymerase’* Gene |
| PREFIX ebola: <<http://bio2rdf.org/ebola_vocabulary>:>  PREFIX drugbank: <<http://bio2rdf.org/drugbank_vocabulary>:> PREFIX dc: <<http://purl.org/dc/terms/>> PREFIX graph: <<http://localhost:8080/ligand_graph>:> PREFIX xsd: <http://www.w3.org/2001/XMLSchema#>  CONSTRUCT {     ?bioUri ebola:chemicalName ?title; ebola:molecularWeight ?molWeight;              ebola:molecularFormula ?formula; graph:pdb-page ?pdbInfo;  graph:drugbank-label ?drugbankLabel; graph:packager ?packagerTitle;  graph:mechanism-of-action ?mechAction; graph:pharmacology ?pharmacologyDesc } WHERE {  ?protein ebola:protein-definition ?proteindefn.     ?protein ebola:hasKeyword ?keyword .     ?keyword ebola:x-pdb ?pdbUri .     ?pdbUri ebola:hasLigand ?bioUri; ebola:pdbPage ?pdbInfo .     ?bioUri ebola:chemicalName ?title; ebola:molecularWeight ?molWeight;              ebola:molecularFormula ?formula; ebola:x-drugbank ?drugbankUri .     ?drugbankUri rdfs:label ?drugbankLabel  FILTER( regex( xsd:string( ?proteindefn ), "polymerase", "is" ) ) .     FILTER( xsd:double( ?molWeight ) < 500 ) .     {         SELECT ?drugbankUri ?mechAction ?packagerTitle ?pharmacologyDesc WHERE {             SERVICE <<http://cu.drugbank.bio2rdf.org/sparql>> {                 ?drugbankUri drugbank:mechanism-of-action ?action;  drugbank:packager ?packager;  drugbank:pharmacology ?pharmacology .  ?action dc:description ?mechAction .  ?packager dc:title ?packagerTitle .  ?pharmacology dc:description ?pharmacologyDesc              }         } GROUP BY ?drugbankUri     } } |
| **Listing 2:** SPARQL CONSTRUCT Query to retrieve the chemical name, molecular formula and PDB Structure information for all ligands with a molecular weight < 500 and associated with the EBOV ‘*Polymerase’* Protein, as well as query the Bio2RDF DrugBank SPARQL endpoint using the SPARQL SERVICE keyword to retrieve the information of action mechanism, pharmacology and packagers for the InChI Key-mapped small molecules. |
| PREFIX ebola:<<http://bio2rdf.org/ebola_vocabulary>:> PREFIX kegg:<<http://bio2rdf.org/kegg_vocabulary>:>  PREFIX dc:<<http://purl.org/dc/terms/>> PREFIX graph:<<http://localhost:8080/ligand_graph>:>  PREFIX xsd: <http://www.w3.org/2001/XMLSchema#>  CONSTRUCT {     ?bioUri ebola:chemicalName ?title; ebola:molecularWeight ?molWeight;              ebola:molecularFormula ?formula; graph:drugbank-label ?drugbankLabel;             graph:kegg-label ?keggLabel; graph:kegg-activity ?activity;  graph:target ?targetLabel; graph:kegg-pathway ?pathwayLabel } WHERE {  ?protein ebola:protein-definition ?proteindefn.     ?protein ebola:hasKeyword ?keyword .     ?keyword ebola:x-pdb ?pdbUri . ?pdbUri ebola:hasLigand ?bioUri .     ?bioUri ebola:chemicalName ?title; ebola:molecularWeight ?molWeight;              ebola:molecularFormula ?formula; ebola:x-drugbank ?drugbankUri .     ?drugbankUri rdfs:label ?drugbankLabel  FILTER( regex( xsd:string( ?proteindefn ), "polymerase", "is" ) ) .     FILTER( xsd:double( ?molWeight ) < 500 ) .     {         SELECT ?drugbankUri ?keggLabel ?activity ?targetLabel ?pathwayLabel WHERE {             SERVICE <<http://cu.kegg.bio2rdf.org/sparql>> {                 ?keggDrugUri kegg:x-drugbank ?drugbankUri .                 ?keggDrugUri rdfs:label ?keggLabel .                  OPTIONAL {?keggDrugUri kegg:activity ?activity} .                 OPTIONAL {?keggDrugUri kegg:target ?target .  ?target rdfs:label ?targetLabel} .                 OPTIONAL {{{?keggDrugUri kegg:pathway ?pathwaymap                     } UNION {?keggDrugUri kegg:str_map ?pathwaymap}} .                     ?pathwaymap rdfs:label ?pathwayLabel}             }         } GROUP BY ?drugbankUri      } } |
| **Listing 3:** SPARQL CONSTRUCT Query to retrieve the Activity, Target and Pathway information from the Bio2RDF KEGG SPARQL Endpoint using the ebola:x-drugbank and kegg:x-drugbank predicates for the interacting ligands with a molecular weight < 500 and associated with the EBOV ‘*Polymerase’* Protein, |
| PREFIX ebola: <<http://bio2rdf.org/ebola_vocabulary>:>  PREFIX pubmed: <<http://bio2rdf.org/pubmed_vocabulary>:>  SELECT DISTINCT ?articleTitle ?abstractText ?journalTitle ?jvol ?jissue WHERE {  ?pdbstructure ebola:hasLigand ?ligand; ebola:x-pubmed ?pubmedArticle .  ?ligand ebola:chemicalName "RIFAMPICIN" .  ?pubmedArticle rdfs:label ?articleTitle;  pubmed:journal ?journalUri ; pubmed:abstract ?abstractUri .  ?abstractUri pubmed:abstract_text ?abstractText .  ?journalUri pubmed:journal_title ?journalTitle .  OPTIONAL {?journalUri pubmed:journal_volume ?jvol}  OPTIONAL {?journalUri pubmed:journal_issue ?jissue}  } |
| **Listing 4:** Retrieving the metadata of scientific publications which provide evidence of the binding of the ligand *‘RIFAMPICIN’* to the EBOV *‘Polymerase’* Protein, for enabling evidence-based hypotheses generation. |

**Appendix II - Knowledge on potential EBOV ‘*Polymerase’* Protein-binding Ligands retrieved from KEGG and DrugBank using the Ebola-Kb endpoint**

**Table 1:** Activity, KEGG Pathway, Target and Packager knowledge for potential EBOV *‘Polymerase’* Protein-binding Ligands retrieved from KEGG and DrugBank using Ebola-Kb endpoint

**Appendix III - Ebola-KB Vocabulary Specification**

**Table 2:** Ebola-KB Vocabulary Specification
